# Effect of Temperature on Breaking of Morphophysiological Dormancy and Seed Germination Leading to Bulblet Production in Two Endemic Tulip Species from Greece

**DOI:** 10.3390/plants12091859

**Published:** 2023-04-30

**Authors:** Elias Pipinis, Stefanos Hatzilazarou, Stefanos Kostas, Rafaela Stagiopoulou, Konstantina Gitsa, Eleftherios Dariotis, Ioulietta Samartza, Ioannis Plastiras, Eleni Kriemadi, Pepy Bareka, Christos Lykas, Georgios Tsoktouridis, Nikos Krigas

**Affiliations:** 1 Laboratory of Silviculture, School of Forestry and Natural Environment, Aristotle University of Thessaloniki, 54124 Thessaloniki, Greece; epipinis@for.auth.gr; 2 Laboratory of Floriculture, School of Agriculture, Aristotle University of Thessaloniki, 54124 Thessaloniki, Greece; hatzilaz@agro.auth.gr (S.H.); skostas@agro.auth.gr (S.K.); konstgitsa@agro.auth.gr (K.G.); 3 Institute of Plant Breeding and Genetic Resources, Hellenic Agricultural Organization Demeter, P.O. Box 60458, 57001 Thessaloniki, Greece; rafaelastagiopoulou@gmail.com (R.S.); eleftheriosdariotis@yahoo.com (E.D.); isamartza@gmail.com (I.S.); 4 Thermokipia Athina, 57500 Epanomi, Greece; info@thermokipiaathina.gr; 5 Laboratory of Systematic Botany, Department of Crop Science, Agricultural University of Athens, Iera Odos 75, 11855 Athens, Greece; ekriemadi@aua.gr (E.K.); bareka@aua.gr (P.B.); 6 Department of Agriculture, Crop Production and Rural Environment, School of Agricultural Sciences, University of Thessaly, 38446 Volos, Greece; chlikas@uth.gr; 7 Theofrastos Fertilizers, Irinis & Filias, Examilia Korithias, 20100 Korinthos, Greece

**Keywords:** botanical tulips, bulbous plants, ecological requirements, RMediterranean biodiversity, R-derived ecological profiling, threatened species

## Abstract

Due to botanical tulips’ economic interest coupled with limited information regarding their seed germination, we investigated the effect of temperature on dormancy release and germination in two endangered local endemic tulip species of Greece (*Tulipa hageri* Heldr., *T. orphanidea* Heldr.). Their germination responses at five constant temperatures (5, 10, 15, 20, and 25 °C) were evaluated in growth chambers, while the type of seed dormancy and the temperature effect on its release were determined based on open-sourced, R-derived species-specific ecological profiles illustrating abiotic conditions of their wild habitats. The results indicated a range-specific temperature dependence in seed germination for both studied species with seed germination observed only in very low temperatures (5–10 °C). The seeds of both species after dispersal had an underdeveloped embryo. The existence of a complex morphophysiological seed dormancy was confirmed in both species by the significant embryo development only at 5 and 10 °C (almost doubled after 30 days) coupled with observed delay in germination only at low temperatures. Furthermore, to facilitate their cultivation and ex situ conservation, the germinated seeds were planted in pots to develop bulblets in greenhouse conditions resulting in bigger *T. orphanidea* bulblets compared to *T. hageri*.

## 1. Introduction

Tulip hybrids are of great significance among ornamental plants utilized for cut flowers, as they are produced and traded on a global scale [1]. Botanical tulips are almost equally famous ornamental plants [1], and Greek botanical tulips among them are associated with a documented global electronic commerce over the internet involving many nurseries in different European countries [2]. To date, wild phytogenetic resources of tulips have gained research interest leading to domestication efforts for different species with the aim to effectively reproduce and cultivate them in artificial environments; such examples have employed to date several Chinese [3,4,5] and Greek [2,6,7] wild-growing tulips. The focus of research efforts toward this direction to date is due to the interesting ornamental characteristics and the unique identity of certain species of botanical tulips, and their strong natural adaptability (even to cultivation settings) coupled with enhanced resilience to pathogens. These assets may trigger—independently or in combination—horticultural and consumer interest, and therefore, botanical tulips can be engaged in coordinated breeding programs aiming to develop new tulip cultivars through exploitation of interesting phytogenetic resources of wild provenance [2,4,5,6].

Among 15 members of the genus *Tulipa* present in Greece either in mainland and/or insular regions [2,6,8], *Tulipa hageri* Heldr. and *T. orphanidea* Heldr. represent two endangered (EN) local endemic species of Greece [2,9]. These two species mainly suffer from modernized agricultural activities, including deep ploughing and widespread application of pesticides and agrochemicals, habitat fragmentation or habitat degradation, land-use or vegetation changes, and seasonal over-collection from the wild. These well-appreciated botanical tulips [2] are wild-growing Greek native tulips found mainly in Sterea Hellas, Peloponnese, and northern Greece [2]. Both species are found in natural habitats, such as xeric Mediterranean phrygana or grasslands *(T. hageri)* and meadows or in agricultural habitats, such as formerly cultivated land in dolines (*T. orphanidea*) or cereal fields and abandoned cultivated lands (*T. hageri*). They both have a wide natural altitudinal range in Greece; *T. hageri* occurs at altitudes between 100 and 1200 m while *T. orphanidea* is observed at altitudes of 700–1600 m [2]. Both species are spring-flowering tulips; *T. hageri*’s flowering season starts in April, and *T. orphanidea*’s flowering season starts one month earlier in March; however, both can be often in bloom till early May [2].

The asexual multiplication of tulip bulbs has been efficiently described in various literature sources (e.g., [10,11,12,13,14]). However, the knowledge about the requirements for seed germination of wild-growing tulip species still remains limited [5,6]. Proliferation-wise, sexual propagation is considered the commonest and most cost-efficient propagation method used in nurseries [15] and is associated with enhanced genetic diversity due to sexual reproduction. However, a major limitation to the sexual propagation of many species is the limited seed germination ability due to either low seed viability and/or inherited mechanisms of seed dormancy [16]. Seed dormancy is a physiological state during which a viable seed fails to germinate even when the environment is favorable for germination [15]. Seed dormancy is a natural adaptation that allows plants to survive in environments characterized by strong seasonality, inducing cellular mechanisms that postpone seed germination until the conditions are suitable for both germination and establishment and survival of the seedlings [17]. Previous studies have highlighted that the seeds of several *Tulipa* species have an underdeveloped embryo after dispersal coupled with a physiological mechanism inhibiting germination, i.e., an established morhophysiological dormancy (MPD) [4,5,18]. There are two requirements for the germination of seeds with MDP: The embryo should develop to a critical size, and the embryo must be released from physiological dormancy. The key to the germination of seeds with MPD is to identify which are the appropriate conditions (especially concerning temperature) that contribute to overcoming dormancy. Multiple levels of MPD have been designated in the literature depending on temperature requirements for physiological dormancy breaking and embryo growth, timing of root and shoot emergence and the response of seeds to gibberellic acid (GA_3_), namely non-deep simple, intermediate simple, deep simple, nondeep simple epicotyl, deep simple epicotyls, deep simple double, non-deep complex, intermediate complex, and deep complex [17]. The abovementioned levels of MPD have been further divided into two general subclasses, i.e., simple MPD where embryo growth takes place at a suitable temperature for warm stratification (≥15 °C) and complex MPD where an embryo grows at a suitable temperature for cold stratification (0 to 10 °C) [17].

Previous studies have shown that seed dormancy of eight wild-growing tulips native in China is classified as non-deep complex MPD [4,5]. However, similar data are absent with regard to *T. hageri* and *T. orphanidea* studied herein, and therefore, the seed dormancy type and their germination requirements have not been investigated to date. Such knowledge would be important for their effective in situ conservation as well as their ex situ propagation for conservation purposes, further enabling simultaneously the coordination of sustainable exploitation strategies. Based on previous literature data [4,5,18], we assumed that the seeds of *T. hageri* and *T. orphanidea* would be dormant due to an undeveloped embryo requiring rather low temperatures to germinate. In this framework, the present study aimed to: (i) examine the effect of incubation temperature on the germination of seeds of *T. hageri* and *T. orphanidea*, (ii) define the type of seed dormancy and investigate how temperature affects its breaking, and (iii) determine an effective germination protocol for the two endangered Greek native tulip species. Furthermore, the size of one-year-old bulblets produced from the seeds of each population per tulip species was evaluated to facilitate future ex situ cultivation in artificial settings. To associate the results of the seed germination experiments with the environmental conditions prevailing in the wild habitats of the studied species, we associated their seed germination responses with respective environmental data (temperature, precipitation). In this way, we tried to unveil the seasonal bioclimatic requirements of each tulip species studied aiming to comprehend their natural life cycle and enable their ex situ conservation.

## 2. Results

### 2.1. Ecological Profiles

**Figure 1:**
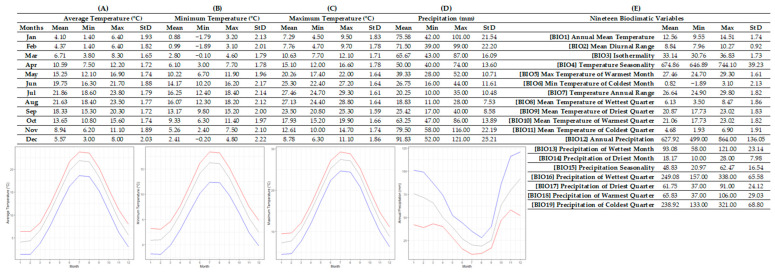
Ecological profile of *Tulipa hageri* based on 12 Greek localities. In this figure, columns represent minimum, maximum, average values, and standard deviation for each one of the sections for: (**A**) the average temperature, (**B**) the minimum temperature, (**C**) the maximum temperature, (**D**) the precipitation, and (**E**) the 19 bioclimatic variables. Line graphs (**A**–**C**) illustrate the minimum (blue), the maximum (red), and the mean (gray) monthly temperature (°C), and the line graph (**D**) illustrates the minimum (red), maximum (blue), and mean (gray) monthly precipitation (mm).

The R-derived ecological profile of *T. hageri* (Figure 1) was assembled on the basis of 12 localities of high accuracy distributed in Peloponnese and north-central Greece. For *T. hageri* sites, the annual average temperature was 12.56 ± 1.74 °C, with the lowest annual average temperature value being observed as 9.55 °C and the highest annual average temperature being 14.51 °C. The average monthly air temperatures in the habitat of this species ranged from 4.10 ± 1.93 °C in January and 4.37 ± 1.82 °C in February, with highest means 21.86 ± 1.79 °C in July and 21.63 ± 1.77 °C in August. The lowest monthly air temperatures in the habitats of the studied localities were recorded in January (0.88 ± 2.13 °C) and February (0.99 ± 2.01 °C), and the single lowest value was −1.90 °C in February. Similarly, the highest monthly air temperatures recorded in the natural habitats of *T. hageri* were in July (27.46 ± 1.61 °C) and August (27.13 ± 1.64 °C), while the highest single temperature was noted in July as 29.30 °C. Average annual precipitation for *T. hageri* sites was 627.92 ± 136.05 mm (lowest annual mean: 499 mm; highest annual mean: 864 mm). The lowest average monthly precipitation was recorded in August (18.83 ± 7.53) as well as in July (20.25 ± 10.48), and the lowest single value was recorded in July (10 mm). The highest monthly precipitation average was recorded in December (91.83 ± 25.21 mm) and November (79.50 ± 22.19 mm), and the single highest value was 121 mm also in December.

**Figure 2:**
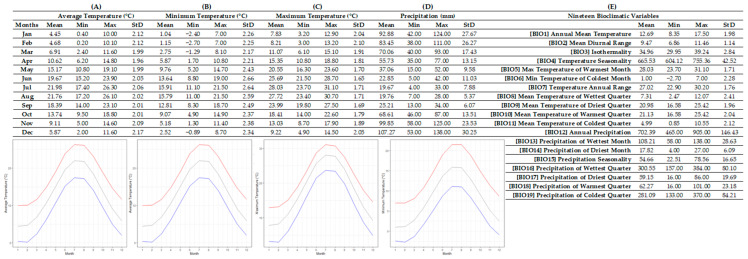
Ecological profile of *Tulipa orphanidea* based on 33 Greek localities. In this figure, columns represent, minimum, maximum, average values, and standard deviation for each one of the sections for: (**A**) the average temperature, (**B**) the minimum temperature, (**C**) the maximum temperature, (**D**) the precipitation, and (**E**) the 19 bioclimatic variables. Line graphs (**A**–**C**) illustrate the minimum (blue), the maximum (red), and the mean (gray) monthly temperature (°C), and the line graph (**D**) illustrates the minimum (red), maximum (blue), and mean (gray) monthly precipitation (mm).

The R-derived ecological profile of *T. orphanidea* (Figure 2) was assembled based on 33 high-accuracy localities of Peloponnese and north-central Greece. For *T. orhpanidea* sites, the annual average temperature was 12.69 ± 1.98 °C, with the lowest annual average temperature value being 8.35 °C and the highest annual average temperature being 17.50 °C. The average monthly air temperatures in the regions that constitute the habitat of this species ranged from 4.45 ± 2.12 °C in January and 4.68 ± 2.12 °C in February (highest mean in July 21.98 ± 2.06 °C and August 21.76 ± 2.02 °C). The lowest monthly air temperatures were recorded in January (1.04 ± 2.26 °C) and February (1.15 ± 2.25 °C), and the single lowest value was −2.70 °C in February. Similarly, the highest monthly air temperatures recorded in the natural habitats of *T. orphanidea* were in July (28.03 ± 1.71 °C) and August (27.72 ± 1.71 °C), while the highest single temperature was recorded in July (31.10 °C). Average annual precipitation for *T. orphanidea* sites was 702.39 ± 146.43 mm (lowest annual mean: 465 mm; highest annual mean: 905 mm). Monthly precipitation prevailing in the abovementioned areas had the lowest average value during July (19.67 ± 7.88) as well as during August (19.76 ± 5.37), and the lowest single value was recorded in July (4 mm). The highest monthly precipitation average was recorded in December (107.27 ± 30.25 mm) and November (99.85 ± 23.53 mm), and the single highest value was 138 mm also in December.

### 2.2. Effect of Incubation Temperature on Seed Germination

A similar seed germination pattern was observed for the two studied tulip species. No germination was observed in seeds of both species incubated at a temperature higher than 10 °C. Germination was only observed in seeds incubated at 5 and 10 °C.

**Figure 3:**
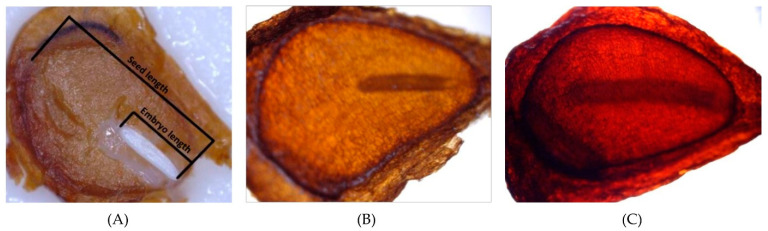
Embryo growth in *Tulipa hageri* seed. Embryo size after seed dispersal (**A**,**B**) and after stratification at 10 °C for one month (**C**).

**Figure 4:**
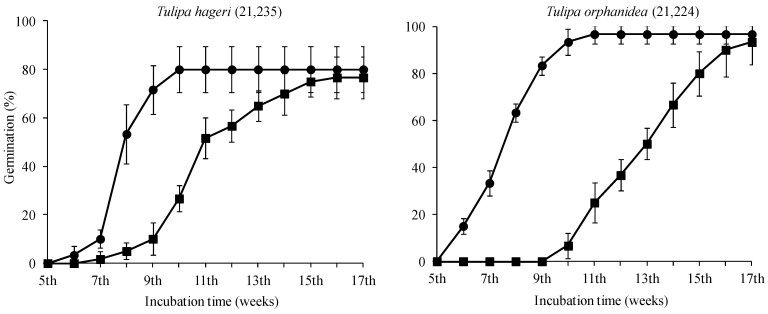
Cumulative germination percentage diagrams of *Tulipa hageri* GR-1-BBGK-21,235 and *Tulipa orphanidea* GR-1-BBGK-21,224 seeds incubated at 5 (●) and 10 °C (■). Bars indicate the standard deviation.

In both species, there were no significant differences between the germination percentages of seeds incubated at 5 and 10 °C (Table 1). In Figure 3, the cumulative germination percentage diagrams at 5 and 10 °C are presented. In both species, the first germinated seeds were recorded at the 6th week, and the germination was completed at the 10th week at the temperature of 5 °C from the onset of the germination test. However, in seeds incubated at 10 °C, the germination started one and four weeks later for the seeds of *T. hageri* and *T. orphanidea*, respectively (Figure 4).

### 2.3. Effect of Cold Stratification and Incubation Temperature on Seed Germination

**Figure 5:**
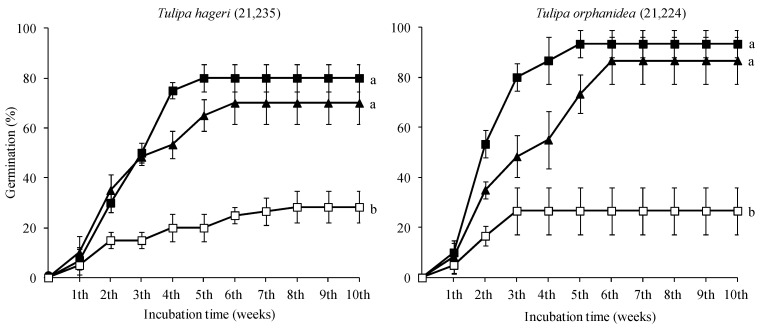
Cumulative germination percentage diagrams of *Tulipa hageri* GR-1-BBGK-21,235 and *Tulipa orphanidea* GR-1-BBGK-21,224 stratified seeds incubated at 10 (■), 15 (▲), and 20 °C (□). In each species, means are statistically different at *p* < 0.05 when not sharing a common letter (comparisons made using the Duncan’s test). Bars indicate the standard deviation.

After cold stratification for one month, seeds of both species germinated at all three incubations temperatures (10, 15, and 20 °C) (Figure 5). However, seeds of both species incubated at 20 °C exhibited the lowest germination percentage, whereas the germination percentages of those incubated at 10 and 15 °C presented no significant differences. The first germinated seeds were recorded after a week from the day that stratified seeds were subjected for germination. The germination was completed at the 5th week at the temperature of 10 °C and a week later at 15 °C from the beginning of the germination test.

### 2.4. Effect of Temperature on Embryo Growth

**Figure 6:**
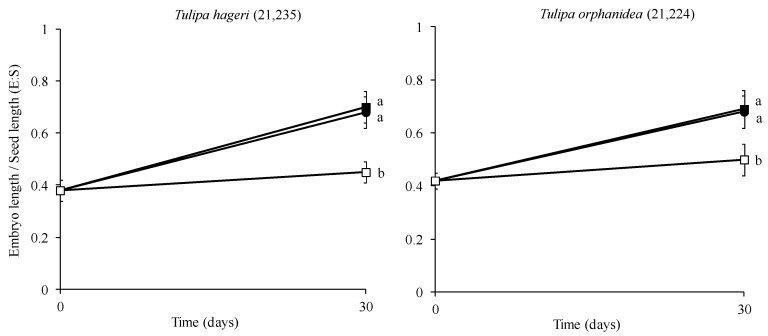
Ratio of mean embryo length to seed length of *Tulipa hageri* GR-1-BBGK-21,235 and *Tulipa orphanidea* GR-1-BBGK-21,224 seeds stratified for 30 days at 5 (●), 10 (■), and 20 °C (□). In each species, means are statistically different at *p* < 0.05 when not sharing a common letter (comparisons made using the Duncan’s test). Bars indicate the standard deviation.

The mean E:S ratio was 0.38 and 0.42 for *T. hageri* and *T. orphanidea* seeds, respectively. The embryo growth of both species was affected significantly by temperature. More precisely, in seeds of *T. hageri*, the E:S ratio at the end of test was the lowest at 20 °C (0.45) (Figure 6), whereas no significant difference was observed between the E:S ratios of seeds stratified at 5 and 10 °C (0.68 and 0.70, respectively). Similarly, in *T. orphanidea*, the lowest value of E:S ratio (0.50) was observed in seeds stratified at 20 °C, whereas no significant difference was observed between the E:S ratios of seeds stratified at 5 and 10 °C (0.68 and 0.69, respectively).

### 2.5. Seedling Production

**Figure 7:**
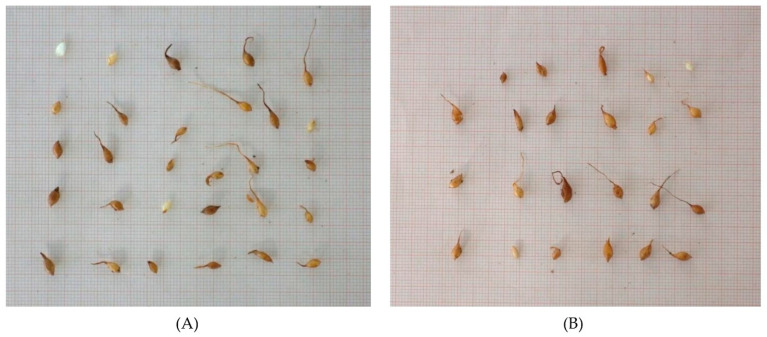
Bulblets produced from seedlings of *Tulipa hageri* (**A**) and *Tulipa orphanidea* (**B**) upon completion of their first growing season.

Upon completion of the first growing period of the seedlings, the means of measurements were 12.10 mg for the mass, 0.187 cm for the length, and 0.398 for the width of the bulblets of *T. hageri*. In *T. orphanidea*, the mass of the bulblets was significantly higher (13.75 mg) (Figure 7, Table 2).

## 3. Discussion

The two Greek tulip species studied showed similar temperature preferences due to similar distribution ranges with higher average annual precipitation recorded for *T. orphanidea* compared to *T. hageri*. Furthermore, similar germination behavior was observed (high germination only at 5 °C and 10 °C). *T. hageri* has been naturally adapted to such temperatures in January (4.10 ± 1.93 °C), February (4.37 ± 1.82 °C), March (6.71 ± 1.65 °C), and April (10.59 ± 1.72 °C) triggering its flowering during April (Figure 4). Similarly, *T. orphanidea* has been adapted to similar temperatures in January (4.45 ± 2.12 °C), February (4.68 ± 2.12 °C), March (10.62 ± 1.96 °C), and April (10.62 ± 1.96 °C), which also match its flowering pattern starting in March (Figure 5).

In general, seeds of any plant species collected from different regions may differ in the degree of dormancy, as inferred by germination percentages of fresh seeds [17]. The results herein clearly showed that temperature affected the germination of seeds, and the seeds of both species germinated only at low temperatures (5 and 10 °C). Further, the increase in temperature to 15, 20, and 25 °C resulted in no seed germination. The optimal temperature for the rate of seed germination of both species was 5 °C (see Figure 4). Similar temperature experiments on seed germination of five Greek tulips have shown that the seeds of *T. australis* Link, *T. bakeri* A.D. Hall, *T. goulimyi* Sealy & Turrill, and *T. clusiana* Redouté may germinate only in a narrow range of very low temperatures (5–10 °C), whereas germination in *T. undulatifolia* Boiss. and *T. goulimyi* may occur at temperatures between 5 and 15 °C [6]. Furthermore, the germination of seeds of the two tulip species studied herein at very low temperatures is in line with previous observations [19,20] regarding the species *T. systola* Stapf and *T. iliensis* Regel, respectively, thus highlighting that a constant temperature of 4–5 °C may induce the highest germination of tulip seeds with no seed germination occurring at temperatures higher than 15–16 °C [4,5].

The temperature response as well as the observed delay in the commencement of germination (6 weeks) designates that the seeds of *T. hageri* and *T. orphanidea* have a type of dormancy. At the time of seed dispersal, the linear-shaped embryo of the seeds in both tulip species was found undersized and underdeveloped (see Figure 3A,B), which implies that it must grow within the seed coat before the radicle emergence. Prior to the experimental test, the seeds of *T. hageri* and *T. orphanidea* exhibited 0.38 and 0.42 embryo-to-seed length (E:S) ratio, respectively. These measurements are consistent with previous studies [4,5], reporting that the ratio of an embryo to seed length in studied Chinese tulips was also less than 0.45 in *Amana edulis* (Miq.) Honda [syn. *Tulipa edulis* (Miq.) Baker)], *T. altaica* Pall. ex Spreng., *T. thianschanica* Regel, *T. sinkiangensis* Z.M. Mao, and *T. iliensis*. It is well established that morphological dormancy is characterized by the presence of an underdeveloped embryo, and in seeds with this type of dormancy, embryo growth and germination occur in about four weeks or less upon favourable conditions, such as moist substrate and suitable temperature [17]. Preceding studies [18] have conveyed that seeds of *T. urumiensis* Stapf possess deep physiological dormancy along with the morphological dormancy. Furthermore, several members of Liliaceae (such as *Tulipa* spp. and members of other genera) set seeds bearing an underdeveloped embryo, thus being associated with either morphological or morphophysiological dormancy [17]. In the present study, after a moist stratification period for 30 days at 5, 10, and 20 °C of *T. hageri* and *T. orphanidea* seeds, the embryo was significantly developed (see Figure 3C). However, the significant increase in embryo length (almost double the ratio of E:S) occurred only at 5 and 10 °C, and specifically, the E:S ratio reached a value of 0.68 in seeds of both species about a week before the onset of germination at 5 °C (see Figure 6). In seeds of both species stratified at 20 °C, the embryo length was increased; however, this ratio was unable to reach the minimum E:S ratio required for germination. Given that the initiation of germination in both tulip species was delayed for at least five weeks, the embryo development was significantly increased in the meanwhile, and germination in both tulip species occurred at low temperatures coupled with negligible germination at warmer temperatures; it can be concluded that the seeds of *T. hageri* and *T. orphanidea* have a complex type of morphophysiological dormancy. However, it is known that the complex morphophysiological dormancy includes three levels: non-deep, intermediate, and deep [17]. Since the seeds of both tulip species need cold stratification for dormancy break and subsequent germination, the non-deep complex morphophysiological dormancy can be ruled out. Nonetheless, it is currently not possible to decipher exactly the level of complex morphophysiological dormancy (intermediate or deep) in the seeds of the two tulips species, and further research is needed to determine the effect of gibberellic acid to seed germination.

After a one-month period of cold stratification, seeds of both species germinated at higher temperatures than those observed in non-stratified seeds. Cold-stratified seeds of both species, which were incubated at 15 °C, germinated at high percentages, equally to those incubated at 10 °C. It is known that cold stratification may reduce the temperature requirement for seed germination [17]. However, the germination percentages of seeds in this study incubated at 20 °C after cold stratification were significantly reduced in both tulip species (see Figure 5). Although a one-month period of cold stratification promoted the breaking of both physiological and morphological dormancy of seeds of both Greek tulip species, they failed to germinate in high percentages when incubated at a high temperature (20 °C). Furthermore, the comparison of the cumulative germination percentage diagrams of *T. hageri* and *T. orphanidea* seeds incubated at 10 °C (Figure 4 compared with Figure 5) may reveal that cold stratification increased the speed of germination. After a 30-day period of moist stratification, the values of the E:S ratio at 5 and 10 °C were similar (see Figure 6); however, the seeds germinated later at 10 °C were compared with those at 5 °C (see Figure 4). Possibly, in seeds incubated at 10 °C, a longer period was required to overcome the physiological dormancy, as it is known that the temperature of 10 °C is usually high to be effective for cold stratification of seeds [7].

The seeds of *T. hageri* and *T. orphanidea* are naturally dispersed during summer months. Both Greek tulip species have developed a seed dormancy mechanism that prevents the germination during adverse conditions or periods when conditions are not favorable and long enough for seedling establishment. The R-derived ecological profiles of the two studied species can provide significant information regarding the climate conditions under which a plant species thrives in its original (natural) habitat. As documented by their respective ecological profiles (see Figure 1 and Figure 2), seeds of both tulip species fail to germinate in autumn because the temperature requirement for physiological dormancy breaking and embryo growth is not fulfilled. Considering that seeds of both tulip species require low temperatures for dormancy induction and germination as well as a long time (6 weeks) for germination to start (further implying that soil needs to stay moist for a long period), the optimum conditions in their natural habitats for seed germination are created in late autumn and during the winter. According to the R-derived ecological profiles (see Figure 1 and Figure 2), it was shown that low temperatures (<10 °C) prevail in late autumn and during the winter in their wild habitats and the increased amounts of precipitation in this period can keep the soil moist enough for seed germination. Therefore, the seeds of these two tulip species are subjected to ideal conditions for dormancy breaking during this period while seed maturation and germination seems to take place by mid-winter with the emergence of seedlings. Several previous studies have documented that the seed germination of species from the Mediterranean region is mainly observed at relatively low temperatures [21,22,23]. This behavior alongside with the results furnished herein for the Greek tulips studied is considered a successful adaption of Mediterranean plants to the seasonally dry environments, ensuring that plants may have a longer period for growing until the typical Mediterranean summer drought.

Tulip hybrids and botanical tulips are usually propagated by bulbs [2], and it is well-known that bulb size affects shoot height, leaf area, and root development [24,25]. However, all tulip bulbs must reach a critical size for flowering to be induced [26]. However, bulb size of botanical tulips is scarcely documented in the literature, and only recently, some studies have provided documentation regarding the growth of one-year-old bulblets produced from seedlings of Greek botanical tulips [6]. *T. orphanidea* bulblets were significantly heavier and longer compared to those of *T. hageri*. The mass, length, and width of the bulblets of *T. australis* are reported to be higher (25.26 mg, 0.245 cm, and 0.491 cm, respectively) compared to the two Greek tulip species studied herein, whereas the respective values regarding *T. bakeri*, *T. goulimyi*, and *T. clusiana* are almost similar [6]. Nonetheless, more research is needed to evaluate the potential genetic variability exhibited by the studied species, and this evaluation will be necessary to determine the possibility of improving the bulb production of the studied species.

## 4. Materials and Methods

### 4.1. Plant Material

**Figure 8:**
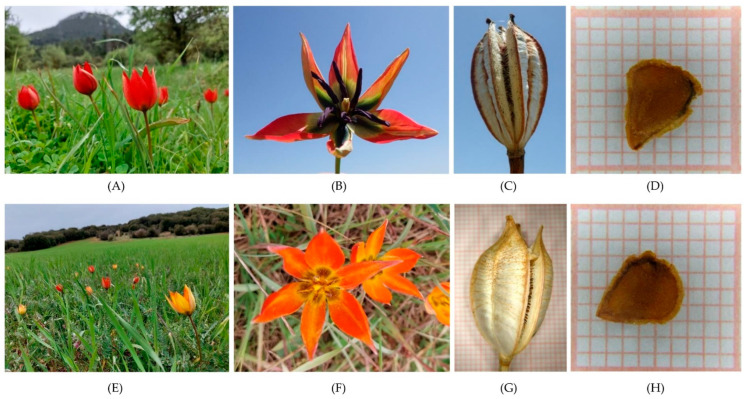
Flowering individuals in original habitats, vertical view of flowers, mature capsules, and seeds of wild-growing *Tulipa hageri* GR-1-BBGK-21,235 (**A**,**B**,**C**,**D**, respectively) and *Tulipa orphanidea* GR-1-BBGK-21,224 (**E**,**F**,**G**,**H**, respectively) collected from Greece.

Capsules with mature seeds from about 10–15 wild-growing individuals of *Tulipa hageri* and *Tulipa orphanidea* were collected by hand before dispersion during a botanical expedition performed in 2021 in the frame of the research project TULIPS.GR (Table 3, Figure 8). The authorized collection was performed using a special permission (64,886/2959 of 6 July 2020; 26,895/1527 of 21 April 2021) issued by the Greek Ministry of Environment and Energy since all wild-growing tulips of Greece are considered protected species covered by the Greek Presidential Decree 67/1981 [2,6]. Upon taxonomic identification of specimens, an IPEN (International Plant Exchange Network) code was provided to each of the collected materials (Table 3). The collected seeds were manually cleaned and were stored dry in glass containers at 3–5 °C before experimentation.

The plant nomenclature of the studied Greek tulip species follows the web version IV of the Flora of Greece—An annotated checklist (https://portal.cybertaxonomy.org/flora-greece/intro, accessed on 3 March 2023).

### 4.2. Distribution Data

To compile the distribution dataset concerning *T. hageri* and *T. orhpanidea* in Greece, we consulted the personal herbaria of Dr. P. Bareka (Department of Crop Science, Agricultural University of Athens, personal communication) and Dr. T. Constantinidis (Department of Biology, National and Kapodistrian University of Athens, personal communication) as well as the unpublished information of the database of the Balkan Botanic Garden of Kroussia, and we further supplemented our data with online data from JAQC herbaria (https://www.jacq.org/, accessed on 9 March 2023) and Lund Virtual Herbarium (http://herbarium.emg.umu.se/, accessed on 9 March 2023). Finally, we cross-checked the dataset with GBIF (https://www.gbif.org/, accessed on 6 March 2023). For this study, we compiled spatial information regarding 12 known localities of *T. hageri* and 33 localities of *T. orphanidea* (Supplementary Material Table S1).

### 4.3. Ecological Data-Mining

Using the abovementioned recorded spatial information, WorldClim’s (version 2.1) environmental data [27], and the R’s raster package [28], we compiled two species-specific ecological profiles by correlating presence points for each species with respective spatial information of the sites where it is known to occur in the wild. The dataset derived from WorldClim consisted of average values for 19 bioclimatic variables as well as monthly precipitation and temperature data. To interlink the datasets, we followed the same approach with previous studies (e.g., [6,29,30,31,32,33,34]). In brief, we created a stack of raster layers with environmental information and extracted numeric information for each one of the overall known presence points of T. hageri and T. orphanidea. We finally organized the extracted point data in a fact sheet for all populations per species, and we further calculated their average, minimum, maximum, and standard deviation values based on the available data, thus summarizing ecological preferences for the two species [6,35,36].

### 4.4. Effect of Temperature on Seed Germination

Germination experiments were started in the middle of January 2022, using the facilities of the Laboratory of Floriculture, School of Agriculture, Aristotle University of Thessaloniki.

**Figure 9:**
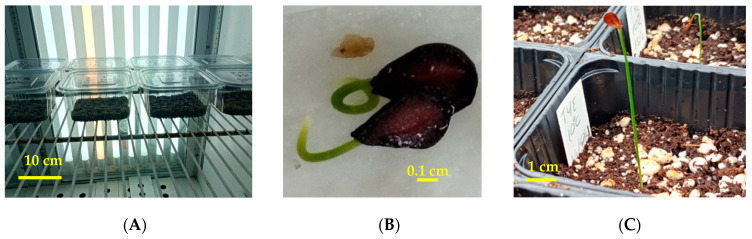
(**A**) Seeds of *Tulipa hageri* and *T. orphanidea* in plastic boxes placed in a growth chamber; (**B**) germinated seeds of *T. hageri* (GR-BBGK-1-21,235); (**C**) seedlings of *T. orphanidea* (GR-BBGK-1-21,224) in pots 20 days after germination.

An independent experiment was performed per tulip species to investigate the effect of temperature on seed germination. The experiment was conducted in controlled growth chambers. Specifically, seeds were incubated at five constant temperatures of 5, 10, 15, 20, and 25 °C with a 12 h daily photoperiod. In each temperature, four replications of 15 seeds were employed. The seeds were placed on moist, sterile sand within 9 × 10 cm transparent plastic containers (Figure 9A) which were randomly arranged in growth chamber shelves, keeping the substrate (sand) moist till the end of the experiment. Every week for four months, all germinated seeds were recorded and then removed from the containers (Figure 9B). A seed was considered germinated on the basis of at least 2 mm long radicle emergence through the seed coat [37].

### 4.5. Effect of Cold Stratification and Incubation Temperature on Seed Germination

For each tulip species, a second germination experiment was carried out to determine the effect of cold stratification on seed germination. The seeds of each species were stratified in 9 × 10 cm transparent plastic containers and were then placed at 5 °C for 30 days. The same substrate as described above was used in plastic containers. In each tulip species, there were 12 plastic containers of 15 seeds. During the seeds’ stratification, the moisture of the substrate in plastic containers was checked intermittently, and water was added whenever necessary to retain its moisture. In both species, no separate control seeds were used due to the limited number of seeds harvested from the wild. However, the untreated seeds (non-cold-stratified seeds) of the previous experiment placed in incubators to germinate can be considered as control seeds. At the end of stratification period, the germination response of seeds at three constant temperatures (10, 15, and 20 °C) was assessed. The 12 randomly divided plastic containers that corresponded to each species were kept with a moist substrate till the end of the germination test and were placed randomly on the shelves of three temperature-controlled growth chambers adjusted to 12 h light/12 h dark photoperiod. All germinated seeds were counted and removed every week for a period of four months. The criterium used for germinated seeds was at least 2 mm long radicle protrusion through the seed coat [37].

### 4.6. Effect of Temperature on Embryo Growth

Another experiment was performed to determine the optimal temperature for embryo growth for each of the two tulip species. Firstly, we took a random sample of 15 seeds from each species to measure in each seed the length of an embryo (E) and a seed (S), allowing us to calculate the ratio E:S (Figure 3A,B). Then, the seeds were subjected to moist stratification at 5, 10, and 20 °C with a 12 h daily photoperiod. In each temperature, a transparent plastic container (9 × 10 cm) with 15 random seeds was placed for each species. The same substrate as described above was used in plastic containers, and it was kept moist as required during the whole experimental period. After 30 days, the length of the embryo and seed was measured in each seed from each temperature per species, and the ratio E:S was calculated (Figure 3C). Finally, the mean E:S ratio and standard deviation were calculated for each sample of 15 seeds. The length of the embryo and seed was measured under a stereoscope equipped with a micrometer.

### 4.7. Seedling Production

The seeds of each tulip species that germinated during the first experiment (Figure 9B) were transferred to small plastic pots (6 × 6 × 6.5 cm dimension) filled with a mixture of enriched peat (TS1, Klassmann) and perlite in a ratio of 3:1. Then, the pots were transferred to an unheated greenhouse where they remained to allow seedling growth. Watering was applied every three days to maintain the proper moisture conditions for seedling growth (Figure 9C).

The seedlings of both tulip species received an integrated nutrient management (INM). The INM fertilization consisted of a nutrient solution, namely PRECISE at 0.6 mL/L, THEOCAL at 0.75 g/L, THEOCOPPER at 3.0 mL/L, and Hyper Phos 570 at 0.3 g/L (Theofrastos fertilizers, Korinth, Greece). Fertilization was started in late March and applied every 15 days until the end of May. In total, five fertilizations were applied and a constant amount of 3 mL of the above nutrient solution was added in each pot per application. The fertilization was usually applied a day after the seedlings’ irrigation. In July 2022, natural desiccation of all the seedlings’ aboveground part, the bulblets were removed from the pots. Subsequently, the measurements of the length and width of each bulblet were performed using a ruler, while its mass was measured using a precision laboratory scale with four decimals.

### 4.8. Statistical Analysis

For each species, a completely experimental design was used. The data were subjected to one-way analysis of variance (ANOVA), and the comparisons of the means were made using the Duncan’s test at a significance level of *p* ≤ 0.05 [38]. Prior to the ANOVA, only the germination percentage data were transformed to arc-sine square root values [39]. Furthermore, the comparison between the two means was made using the *T* test [38]. All statistical analyses were carried out using SPSS 21.0 (SPSS, Inc., Chicago, IL, USA).

## 5. Conclusions

In this study, R-derived informative ecological profiles for *T. hageri* and *T. orphanidea* were outlined, thus providing insight for the first time into the abiotic environmental conditions constituting the natural habitats and shaping the ecological niches of *T. hageri* and *T. orphanidea*. Furthermore, the type of seed dormancy of both studied species was documented, and their germination requirements are firstly reported herein along with data regarding the initial development of bulblets. The results of the present study indicated the existence of a complex morphophysiological dormancy and the range-specific temperature dependence for the germination of seeds of both wild-growing Greek tulips. The effective sexual propagation protocol developed herein can facilitate both in situ conservation efforts and ex situ conservation actions for the two endangered local endemic tulip species of Greece, allowing at the same time their sustainable exploitation as ornamental plants.

## Figures and Tables

**Table 1:** Mean germination percentages and standard deviation of *Tulipa hageri* and *Tulipa orphanidea* seeds incubated at 5 and 10 °C.

	Incubation Temperature	
Greek Tulip IPEN Accession Number	5 °C	10 °C
*Tulipa hageri* GR-BBGK-1-21,235	80.00 a * ± 9.43	76.67 a ± 8.61
*Tulipa orphanidea* GR-BBGK-1-21,224	96.67 a ± 3.85	93.33 a ± 9.43

* Means in the same row are statistically different at *p* < 0.05 when not sharing a common letter.

**Table 2:** Mass (mg), length and width (cm) of bulblets produced by seeds from *T. hageri* and *T. orphanidea* after the first period of growing. Means ± standard error values are given.

Greek Tulip IPEN Accession Number	Bulblet Mass (mg)	Bulblet Length (cm)	Bulblet Width (cm)
*Tulipa hageri* GR-BBGK-1-21,235	12.10 b * ± 0.39	0.187 a ± 0.009	0.394 a ± 0.013
*Tulipa orphanidea* GR-BBGK-1-21,224	13.75 a ± 0.55	0.181 a ± 0.011	0.438 a ±0.021

* Means in the same column are statistically different at *p* < 0.05 when not sharing a common letter.

**Table 3:** Seed collection details and IPEN (International Plant Exchange Network) accession numbers of seedlots of *Tulipa hageri* and *Tulipa orphanidea* in WGS84 coordinates system.

Scientific Name	IPEN Accession Number	Altitude (m)	Collection Site and Area	Collection Date	Latitude (North)	Longitude (East)
*Tulipa hageri*	GR-BBGK-1-21,235	569	Sochos, Thessaloniki	29 June 2021	40.799025	23.389888
*Tulipa orphanidea*	GR-BBGK-1-21,224	800	Kampos Karyon, Arkadia	20 June 2021	37.308444	22.420063

## Data Availability

All data supporting the results of this study are included in the manuscript, and the datasets are available upon request.

## Supplementary Materials

The following supporting information can be downloaded at: https://www.mdpi.com/article/10.3390/plants12091859/s1, Table S1. Spatial (geo-referenced) information in a WGS84 coordinates system of voucher and/or living specimens of *Tulipa hageri* and *T. orphanidea* localities in Greece, compiled from the database of the Balkan Botanic Garden of Kroussia, the personal herbaria of Dr. P. Bareka (Department of Crop Science, Agricultural University of Athens), and Dr. T. Constantinidis (Department of Biology, National and Kapodistrian University of Athens) after supplementation with respective data retrieved and elaborated from Lund Virtual Herbarium (http://herbarium.emg.umu.se/, accessed on 31 March 2023) and JAQC herbaria (https://www.jacq.org/, accessed on 31 March 2023) with respective cross-checking in the GBIF portal (https://www.gbif.org/, accessed on 31 March 2023).
